# The Effect of One-Year Fermentation of *Maesil* Fruit (*Prunus mume*) Sugar Syrup on Amygdalin Level: A Natural Toxic Compound

**DOI:** 10.3390/foods13162609

**Published:** 2024-08-20

**Authors:** Srinivasan Ramalingam, Vishal Kumar, Ashutosh Bahuguna, Jong Suk Lee, Myunghee Kim

**Affiliations:** 1 Department of Food Science and Technology, Yeungnam University, Gyeongsan 38541, Republic of Korea; sribt27@gmail.com (S.R.); vishalkumar@yu.ac.kr (V.K.); ashubahuguna@ynu.ac.kr (A.B.); 2 Department of Food & Nutrition & Cook, Taegu Science University, Daegu 41453, Republic of Korea; jslee1213@ynu.ac.kr; 3 Research Institute of Cell Culture, Yeungnam University, Gyeongsan 38541, Republic of Korea

**Keywords:** amygdalin, cyanoglycoside, fermentation, *maesil*, *Prunus mume*, sugar syrup

## Abstract

*Prunus mume* (*maesil*) is an economically important fruit in Korea. Recently, public interest in *maesil* sugar syrup is increasing. However, the presence of toxic amygdalin in the fruit syrup is a concern. Thus, the current investigation aimed to observe effects of *maesil* maturity, ripening methods, processing, and fermentation period on the amygdalin level in *maesil* sugar syrup. Six different types of *maesil* sugar syrup were prepared and amygdalin content was monitored at 3-month intervals. Higher levels (>63 mg/L) of amygdalin were found in syrups prepared from unripe fruit compared to those in syrups made from ripe fruit after 3 months of fermentation. A rapid reduction in amygdalin content was observed until 9 months in all syrups, gradually reducing to <5 mg/L at 12 months. More than 9 months of maturation is crucial for reducing the amygdalin content *maesil* sugar syrup, regardless of fruit maturity, source of fruit, and processing method.

## 1. Introduction

*Prunus mume* is an important fruit crop owing its health benefits and characteristic sensory attributes [1]. The common Korean name for the fruit of *P. mume* is *maesil*, which is also known as Japanese apricot or Chinese plum. *Maesil* is used to prepare various value-added products such as pickles, squashes, flavoring agents, alcoholic beverages, and extracts for medicinal use in Korea, China, and Japan [2,3]. Fermented *maesil* products are gaining popularity and their consumption rate has increased in Korea because of their nutritional and health benefits like antioxidant and anti-osteoporosis activities [4].

In general, *maesil* is not consumed as raw fruit owing to its strong sour taste and the presence of a toxic cyanogenic glucoside D-mandelonitrile-β-D-gentiobioside (amygdalin) [5]. During physical damage such as chewing and bruising of *maesil*, enzymatic degradation of amygdalin occurs, producing hydrogen cyanide (HCN) and a ketone or aldehyde [6]. Consumption of cyanogenic material from plant-based foods has been reported to cause both acute and sub-acute health issues, such as abdominal cramps, cardiac arrest, failure of the circulatory and respiratory system, coma, dizziness, headaches, mental confusion, nausea, vomiting, weakness, and death in extreme conditions, depending on the dosage [7].

Currently there is growing public concern about the toxicity of *maesil* products. In particular, the toxicity of *maesil* sugar syrup is receiving substantial public attention because of the significant amounts being consumed every day [2]. *Maesil* sugar syrup is prepared by soaking unripe *maesil* in sugar for a long time (more than 3 months) at room temperature for natural fermentation. The seeds of unripe *maesil* contain a high amount of amygdalin, which raise public concern to use these fruits for the sugar syrup preparation [8]. It is believed that using either ripe or destoned fruits, whether ripe or unripe, in the production of *maesil* sugar syrup, along with prolonging the fermentation period, will lower the level of amygdalin in the finished product. However, the influence of ripe, unripe, and destoned fruits, and of the fermentation period on the amygdalin content in the *maesil* sugar syrup has not been thoroughly studied. Therefore, the present investigation aims to evaluate the effect of *maesil* maturity, ripening methods, processing, and fermentation period on the amygdalin content in *maesil* sugar syrup.

## 2. Materials and Methods

### 2.1. Fruit Source and Maesil Sugar Syrups Preparation

**Figure 1:**
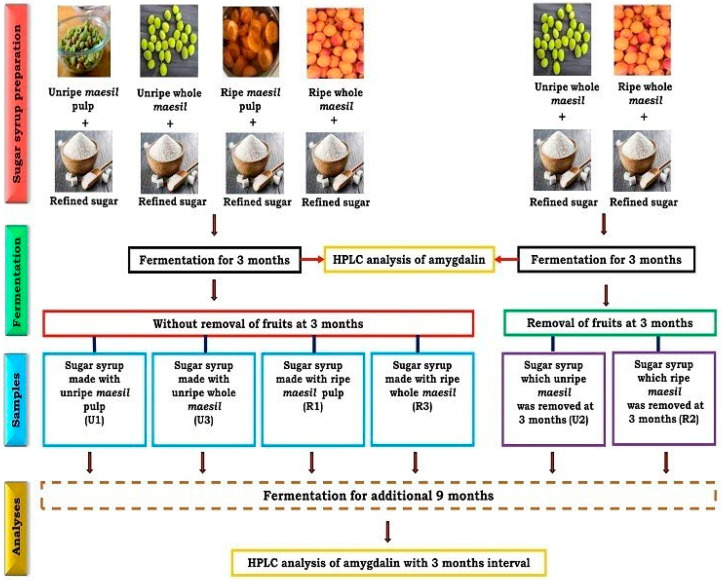
A ray diagram represents the preparation of different *maesil* sugar syrups and timeline of sampling and amygdalin estimation in *maesil* sugar syrups.

Ripe and unripe fruits of the *P. mume* (namgo variety) were procured from an orchard located in Cheongdo-gun, Gyeongsangbuk-do, Republic of Korea. The process of preparing and fermenting *maesil* sugar syrups has been detailed in our prior literature [9]. Briefly, equal weights of various fruit samples, including whole fruits (two batches) and destoned fruits (pulp only) of both ripe and unripe *maesil*, were separately stacked in sterilized glass jars in alternating layers (1–2 cm) with equal volumes of refined sugar. The bottom and top layers (1–2 cm) consisted of sugar, and the jars were sealed with lids. These jars were left to undergo fruit leaching (due to osmosis) and fermentation at room temperature for 3 months. After this 3-month fermentation period, the fruit material was removed from one batch each of the ripe and unripe whole-fruit *maesil* sugar syrups, and fermentation continued with the other samples. Samples were collected at 3-month intervals for amygdalin estimation until 1 year (Figure 1). After 3 months, we obtained six types of *maesil* sugar syrup: sugar syrup made using unripe *maesil* pulp (U1), sugar syrup from which unripe *maesil* was removed after three months (U2), sugar syrup made using unripe whole *maesil* (U3), sugar syrup made using ripe *maesil* pulp (R1), sugar syrup from which ripe *maesil* was removed after three months (R2), and sugar syrup containing ripe whole *maesil* (R3) [9]. The preparation method, sampling, and amygdalin estimation periods are illustrated in Figure 1. Additionally, the seeds, seed coat (testa), seed shell (endocarp), and fruit pulp with skin (mesocarp along with exocarp) of both ripe and unripe *maesil* used to make *maesil* sugar syrups were separated, vacuum freeze-dried, mechanically pulverized, and stored at −20 °C until further analysis. These vacuum freeze-dried parts of both ripe and unripe fruits (the primary starting raw materials for the sugar syrups) were used for the estimation of amygdalin.

### 2.2. Extraction of Amygdalin

The extraction of amygdalin from the *maesil* sugar syrups and plant samples was carried out by using the method of Kim et al. (2010) [10]. In short, 100 mL of methanol and about 1 mL of *maesil* sugar syrup were mixed together and extracted for 1 h at 37 °C in a shaking water bath. The extract was concentrated under vacuum at 45 °C after being filtered through Whatman filter paper (No. 1). The residue was dissolved in 50 mL of triple-distilled water and mixed with 50 mL of hexane for liquid-liquid extraction in a separation funnel. The hexane fraction was collected and discarded. Fifty milliliters of ether were used to extract the aqueous fraction again, and the ether fraction was discarded. The aqueous fraction was then concentrated at 45 °C with a lower pressure. For HPLC analysis, the concentrated residues were filtered through a 0.2 µm syringe filter after being dissolved in 5 mL of triple-distilled water. Similarly, 0.1 g of freeze-dried, powdered plant samples seeds, seed coat (testa), seed shell (endocarp), and pulp (mesocarp along with exocarp) were extracted for the determination of their amygdalin content using the above procedure. All experiments were conducted in triplicate.

### 2.3. Estimate of the Amygdalin

Bolarinwa et al. (2014) method was used to measure the amygdalin content of the *maesil* sugar syrups and plant samples [5]. An HPLC system with a UV-visible detector (Dionex UltiMate 3000 UHPLC+ focused, Thermo Fisher Scientific, Waltham, MA, USA) was used to quantify amygdalin. The amygdalin in the HPLC was separated employing a reversed-phase C18 octadecyl-silica column (5 µm pore size, 4.6 × 250 mm, chemcobond 5-ODS-H, K3A96, ChemcoPlus Scientific Co., LTD, Osaka, Japan). The mobile phase of the HPLC consisted of 75% water and 25% methanol, maintained for 20 min at an isothermal temperature of 40 °C, with a flow rate of 1 mL/min. Samples (20 µL) were automatically injected from 1 mL HPLC sampling vials loaded into the Thermo Scientific Dionex UltiMate 3000 Well Plate Autosampler, with the system temperature kept at 4 °C.

Analytical grade amygdalin (99% purity) was obtained from Sigma-Aldrich (St. Louis, MO, USA). A stock solution was prepared by dissolving 10 mg of amygdalin in 10 mL of sterile triple distilled water. This stock solution was then diluted to create standard amygdalin concentrations of 2, 5, 10, 20, 40, and 50 mg/L for HPLC analysis, which were used to plot the standard curve for sample quantification. The limit of detection (LOD) and limit of quantitation (LOQ) were determined using the standard plot of amygdalin. The detected amygdalin content from fruit samples and sugar syrups was represented in g/kg of dry weight and mg/L, respectively.

### 2.4. Statistical Analysis

Each of the amygdalin measurement was carried out thrice or more, and the findings were expressed as a mean ± standard deviation. Software from IBM (Chicago, IL, USA) called SPSS Statistics 23 was used for statistical analysis. Using a 95% confidence level at *p* < 0.05, one-way analysis of variance (ANOVA) in a fully randomized design, along with either a *t*-test or Duncan’s multiple range test, was employed to identify significant differences between the samples.

## 3. Results and Discussion

**Figure 2:**
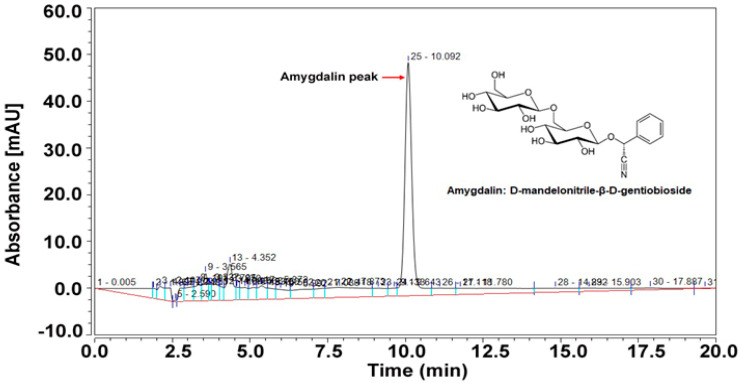
Chromatogram of standard amygdalin (50 mg/L) obtained from the high-performance liquid chromatography (HPLC). Inside the chromatogram, the molecular configuration represents the structure of amygdaline.

Standard amygdalin (50 mg/L) appeared at a characteristic retention time of approximately 10.09 min using 25% methanol mobile phase at 214 nm in the HPLC analysis (Figure 2). The HPLC instruments have limit of detection (LOD) 0.3516 mg/L and limit of quantification (LOQ) 1.0655 mg/L. Under the analyzed HPLC instrument conditions, amygdalin content was detected in both the sugar syrup and different parts of *maesil*. The chromatograms of the samples (Figures S1–S6) contained a few other unknown peaks, which were attributed to other chemicals in the samples. A wide range of amygdalin content (from 0 to 52.85 g/kg) was detected in different parts of the *maesil* with maximum amount in unripe *maesil* seeds (52.85 ± 2.00 g/kg), followed by that in ripe *maesil* seeds (40.33 ± 3.96 g/kg) (Table 1). Similarly, unripe fruit pulp contained a significantly higher amount of amygdalin (0.81 ± 0.22 g/kg) than ripe fruit pulp (0.19 ± 0.01 g/kg). The seeds and fruit pulp of unripe fruits contained 98.48% and 1.52% of the amygdalin content, respectively. Similarly, the seeds and fruit pulp of ripe fruits contained 99.52% and 0.48% of the amygdalin content, respectively. Interestingly, no presence of amygdalin was detected in seed shell (endocarp) and seed coats (testa) of both ripen and unripen *maesil* (Table 1). These results indicate that seeds are the major source of amygdalin in both ripe and unripe *maesil*. Previous studies have reported higher amounts of amygdalin in seeds (ranging from 100–340 mg/100 g) compared to fruit pulp (ranging from 5–35 mg/100 g) in various cultivars of *P. mume*, supporting these findings (Son et al., 2017) [8]. The amygdalin content in seeds and pulp of ripe fruit was 23.70% and 76.02% lower than that of unripe fruit, consistent with the findings of Son et al. (2017) [8]. Interestingly, amygdalin was not detected in either ripe or unripe seed coat and seed shell. Similarly, the fruit enlargement stage of *Hikawa hakuho* variety peach showed no amygdalin content in the seed shell [11]. It should be noted that the seed shell is made of fibrous lignin and composes the hard part of the fruit, which may affect the extraction of amygdalin by restricting the transport through the seed shell [12].

**Figure 3:**
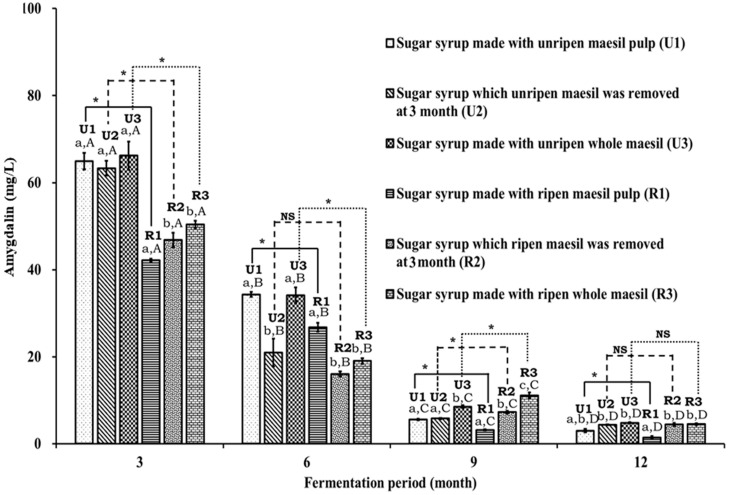
Total amygdalin concentration (mg/L) in *maesil* sugar syrups (derived from mixture of unripe and ripe fruit) over a 1-year fermentation period. Each value in the bar graph represents the mean ± SD of three independent experiments. *: Significant difference between syrups made from ripe (R) and unripe (U) fruits at specific time points; NS: represents non-significant difference between the groups. Lowercase alphabets (a–c) and uppercase alphabets (A–D) indicate significant differences within each group [unripen (U) and ripen (R)] and between the same sample at different time intervals compared to initial values, respectively. The *p* < 0.05 was obtained from the One-way ANOVA followed by Duncan’s multiple range test.

The results of the long-term amygdalin analysis of all *maesil* sugar syrups revealed that the amygdalin content decreased during fermentation (Figure 3). A higher amount of amygdalin (63.31 ± 1.68 to 66.19 ± 3.21 mg/L) was found in *maesil* sugar syrups prepared with unripe *maesil* (U1–U3), compared with that found in syrup prepared with ripe *maesil* (R1–R3) (42.17 ± 0.30 to 50.39 ± 0.84 mg/L), after three months of fermentation. The lowest amount of amygdalin (42.17 ± 0.30 mg/L) was detected in R1 sample. A significantly (*p* < 0.05) ~1.5-fold higher prevalence of amygdalin was detected in *maesil* sugar syrups prepared with unripe *maesil* pulp (U1) compared to those prepared with ripened *maesil* pulp (R1) after three months of fermentation (Figure 3). Consistently, a significant (*p* < 0.05) ~1.3-fold higher concentration of amygdalin was detected in *maesil* sugar syrups with unripe *maesil* (U2 and U3), compared to the similar sugar syrups prepared with ripened *maesil* (R2 and R3), respectively (Figure 3).

Visual observations of the textural integrity of both ripe and unripe processed *maesil* indicated that osmotic stress induced by excessive sugar had a negative impact. This stress led to irregularities such as texture damage to the fruit skin and partial or complete liquefaction of the fruit pulp. Although neither ripe nor unripe whole fruits underwent liquefaction, they did experience shrinkage due to osmotic stress and water loss. When the seeds were extracted, the fruit was halved, increasing the surface area exposed directly to sugar. The absence of fruit skin on the cut side may have resulted in textural instability, which was visually evident in the deseeded fruit material of the R1 sample.

Due to its unique physical properties, the unripe fruit pulp maintained its texture better than the ripe fruit pulp [3,13]. Consequently, no significant changes in structural integrity (except shrinkage) were observed visually in the unripe *maesil*, nor in their amygdalin content in the U1–U3 sugar syrups. The observed differences in amygdalin concentration between unripe (U1–U3) and ripe (R1–R3) fruit sugar syrups may be attributed to variations in the original fruit’s amygdalin content, the rate of amygdalin release from the fruit to the *maesil* sugar syrups, and the fermentation process.

The findings of the 6-month amygdalin analysis showed a remarkable (47–66%) decrease in the amygdalin content in all tested samples as compared to the amygdalin content observed at 3-month (Figure 3). The maximum 67% and 66% amygdalin reduction was observed in sample U2 (20.94 ± 3.21 mg/L), and in sample R2 (16.06 ± 0.57 mg/L), which are the sugar syrups from where *maesil* was removed after three months of fermentation. Sugar syrup samples U1 and U3 had the highest amounts of amygdalin of 34.32 ± 0.54 mg/L and 34.12 ± 1.85 mg/L, indicating a 47.12% and 48.45% reduction of amygdalin, respectively, compared with the corresponding samples fermented for 3 months. The maximum amount of amygdalin among the ripe *maesil* sugar syrups (R1–R3) was noticed in sample R1, with the lowest percentage reduction (36.46%) of amygdalin in the 6-month fermented samples. Sample R3 contained 19.03 ± 0.64 mg/L of amygdalin, corresponding to a 62.23% decline in amygdalin content when compared with the sample fermented for 3 months.

The significant differences between the amygdalin content of the sugar syrup samples are due to different sources of fruit, the rate of amygdalin release, and microbial action during fermentation. The lack of a fruit source in U2 and R2 resulted in no liberation of amygdalin to the *maesil* sugar syrup, which is a probable reason for the significant reduction in amygdalin content. The loss of toughness and liquefaction of the fruit tissue and cell structure of ripe *maesil* in R1 might be the reason for the high amount of amygdalin release [6].

Moreover, all tested sugar syrup samples were found to contain 3.8–4.4 log CFU/mL of sugar-tolerant microbes, specifically *Zygosaccharomyces rouxii* [9]. *Z. rouxii* is the primary microorganism present in all *maesil* sugar syrups, leading to alcohol fermentation [9]. During this fermentation, these microbes utilize amygdalin for their growth, which might explain the reduction of amygdalin in all the *maesil* sugar syrups compared to the samples fermented for 3 months [14].

In general, the enzymatic hydrolysis of amygdalin by glycosidases and hydroxyl nitrile lyases results in the production of glucosides, HCN, and ketone/aldehyde as a defensive mechanism against insect infestation in plants [15]. The isolated *Z. rouxii* in the *maesil* sugar syrups possess both β-glycosidases and hydroxynitrile lyase enzymes, which are capable of hydrolyzing amygdalin into benzaldehyde and HCN [14]. HCN, a toxic hydrolysis product of amygdalin, either evaporates due to its high volatility or is utilized by *Z. rouxii* through mineralization as a source of carbon and nitrogen, or is converted into ammonia and carbon dioxide [14,16]. Son et al. (2017) reported that the concentration of amygdalin in *maesil* sugar syrups declined after fermentation of three months [8], supporting our findings.

The results of the 9-month analysis revealed a significant reduction in the amygdalin quantity of all tested sugar syrup samples (Figure 3). The lowest amygdalin content was observed in R1 (3.18 ± 0.13 mg/L), representing a 92.44% and 88.10% reduction compared to the 3- and 6-month samples, respectively. U1 and U2 contained amygdalin (5.61 ± 0.17 mg/L and 5.82 ± 0.13 mg/L) that represented 91.36% and 90.80% and 82.66% and 72.20% decrease in amygdalin content, compared with 3- and 6-months fermented samples, respectively. The whole fruit *maesil* sugar syrups (U3 and R3) possessed the highest levels of amygdalin in both unripe and ripe fruit *maesil* sugar syrups. Compared to the total amygdalin level of the samples fermented for 3 and 6 months, a reduction by 87.08% and 74.94% was observed in U3, whereas a reduction by 77.92% and 41.53% was observed in R3, respectively. The R2 *maesil* sugar syrup contained 7.25 ± 0.32 mg/L of amygdalin, which decreased to 84.50% and 54.81% in samples fermented for 3 and 6 months, respectively.

The low level of amygdalin found in the samples fermented for 9 months might be due to the peak release rate of amygdalin occurring at three months. After this period, there was little to no further release, combined with a significant rate of degradation, which lowered the amygdalin content in all *maesil* sugar syrups [8]. Consequently, slightly higher amounts of amygdalin were detected in U3 and R3, where the fruit source remained present throughout the fermentation process.

The present results revealed that the amygdalin content in the fruit pulp (0.4–1.5% of the total; 0.2–0.8 g/kg of dry weight) was much lower than that in the seeds (>99–98% of the total; 42–53 g/kg of dry weight), which is another possible reason for the low amygdalin levels. A decline in the quantity of amygdalin over time indicates that the amygdalin content in *maesil* sugar syrups may have been predominantly derived from the fruit pulp and not from the seeds. The tough, fibrous, lignin-based endocarp (seed shell) may prevent the seeds from osmotic damage stress [17], resulting in no migration of amygdalin from the seeds to the syrup. Thus, the quantity of amygdalin gradually decreases over time in *maesil* sugar syrups.

After 12 months of fermentation, all the sugar syrup samples showed more than a 90% decrease in the amygdalin content compared to that in 3-month samples, and at least a 25% reduction compared to the samples fermented for 9 months (Figure 3). The *maesil* sugar syrups prepared from the deseeded fruit in both ripe and unripe *maesil* sugar syrups contained a lower amygdalin content than all other *maesil* sugar syrups. The lowest amygdalin content (1.45 ± 0.29 mg/L) was found in R1, which also had the greatest decrease in amygdalin content, at 96.54% and 54.26% for the 3- and 9-month samples, respectively, followed by U1 (2.99 ± 0.40 mg/L) with a 95.38% decrease in the amygdalin level compared to the 3-month U1 sample. U2, U3, R2, and R3 contained nominal amounts of amygdalin: 4.37 ± 0.09, 4.82 ± 0.12, 4.45 ± 0.34, and 4.49 ± 0.22 mg/L, respectively, which are not significantly different. Compared with the 3-month fermented samples, amygdalin reduction of 93.09%, 92.72%, 90.49%, and 91.07% was found in U2, U3, R2, and R3, respectively. The amygdalin content decreased steadily in both ripe and unripe *maesil* sugar syrups between 3 and 12 months, and the rate was similar for ripe and unripe *maesil* sugar syrups. The outcome of other *maesil*-based studies revealed that a gradual degradation of amygdalin occurs after 3 months to 1 year of the fermentation process [18], which supports the present findings.

The decrease in amygdalin concentration from 9 months (3–12 mg/L) to 12 months (1–5 mg/L) was relatively minor compared to the more substantial reduction observed from 6 months (16–35 mg/L) to 9 months (3–12 mg/L). Despite this smaller absolute reduction (in mg/L), the relative decrease in amygdalin content between 9 and 12 months was still significant, ranging from 24% to 60% (Figure 3). A possible reason for this could be the low concentration of amygdalin in the syrup relative to the number of microbes involved in its degradation. These microbes may require a minimum threshold level of amygdalin to initiate their degradation process. When the amygdalin concentration falls below this threshold, the microbes might switch to utilizing other substrates present in the *maesil* sugar syrups. Consequently, microbial growth could continue to increase throughout the study period in all *maesil* sugar syrups. This is supported by our previous report, which demonstrated that the growth of *Z. rouxii*, the primary microbe involved in *maesil* sugar syrup fermentation, increased from 3.6 log CFU/mL at 3 months to 5.6 log CFU/mL at 12 months [9].

The present results show that the amygdalin content of all *maesil* sugar syrups was less than 5 mg/L at the end of 1 year of fermentation, which is at least 27-fold lower than that reported by Go et al. (2018), who revealed 134.98 ± 5.96 mg/L and 166.82 ± 4.16 mg/L amygdalin content in *maesil* sugar syrups with and without fruit, respectively [2]. The difference in amygdalin concentrations of the *maesil* sugar syrups might be due to variations in the *P. mume* cultivar, fermentation environment, microflora, fruit maturity and processing method and is supported by the findings of Son et al. (2017) [8]. Although Go et al. (2018) reported a higher quantity of amygdalin (134.98 to 166.82 mg/L) compared to the levels found in the present study (63 to 67 mg/L), they noted that this concentration of amygdalin was not toxic to rats [2]. According to EFSA (European Food Safety Authority), the complete degradation of 1 g amygdalin can produce 59 mg of HCN [19]. That mean theoretically, approximately ~17 mg/L amygdalin will produce 1 mg of HCN. In this way, the theoretical yield of HCN from the detected amygdalin quantities (>17 mg/L) in the 9- and 12-month fermented *maesil* sugar syrups in our study is below the threshold for acute and chronic toxicity to humans (>1 mg/kg of HCN) as per described by FSANZ (Food Standards Australia New Zealand) [20]. There is currently no global regulatory entity that has established a maximum allowable limit for amygdalin in food for human consumption. The toxicity of amygdalin, resulting from the release of HCN, can vary depending on individual susceptibility. In general, *maesil* sugar syrups are consumed after a 10-fold dilution with water. Thus, it is assumed that our 3- and 6-month fermented *maesil* sugar syrups would not cause toxicity after dilution. An adequate reduction (87.35%) in amygdalin content was attained at the 9th month for the overall (both ripe and unripe) *maesil* sugar syrups with the least amygdalin content (<13 mg/L). Thus, the present study suggests that a minimum of 9 months of fermentation of *maesil* sugar syrups should be conducted, both for ripe and unripe *maesil*, to deliver a good traditional taste with low amygdalin content.

## 4. Conclusions

It can be concluded from this study that a higher amount of cyanogenic glycoside amygdalin is present in unripe *maesil* than in ripe *maesil*. In particular, seeds contain more than 98.42% of the amygdalin in both ripe and unripe *maesil*. Significantly higher quantities of amygdalin (>63 mg/L) were observed in all *maesil* sugar syrups prepared from unripe *maesil* than in those prepared from ripe *maesil* after 3 months of fermentation. Thereafter, the amygdalin content in all *maesil* sugar syrups gradually decreased until the end of 1 year of fermentation. A rapid reduction (up to 87%) of amygdalin was observed up to the 9th month of fermentation. After this, a low rate of reduction (5.87%) was found in all samples, regardless of fruit maturity and fruit processing. The present findings demonstrate that all prepared *maesil* sugar syrups were non-toxic (<17 mg/L calculated value of amygdalin) for consumption after a minimum of 9 months of fermentation, irrespective of fruit maturity (ripe and unripe *maesil*), source of fruit material (whole fruit or destoned fruit), and processing methods (fermentation with *maesil* fruit or with removal of *maesil* fruit at 3 months). In addition, a long maturation period (since 3–9 months is a crucial period for the reduction of amygdalin content) ensures the removal of most of the amygdalin and imparts a unique flavor and taste to the *maesil* sugar syrups. Further studies are in process to identify the mechanism of amygdalin reduction by native microorganisms present in *maesil* sugar syrups.

## Figures and Tables

**Table 1:** Amygdalin content in different parts (pulp with skin, seed shell, seed coat, and seed) of unripe and ripe *Prunus mume* fruit (*maesil*).

*Maesil* Parts	Amygdalin in Unripe *maesil* (g/kg of Dry Weight)	Amygdalin in Ripe *maesil* (g/kg of Dry Weight)
Pulp with skin (mesocarp with exocrap)	0.81 ± 0.22 ^a^*	0.19 ± 0.01 ^b^
Seed shell (endocarp)	Not detected	Not detected
Seed coat (testa)	Not detected	Not detected
Seed	52.85 ± 2.00 ^a^	40.33 ± 3.96 ^b^

* Different superscript letters (a and b) within each row indicate significant differences (*p* < 0.05) based on the *t*-test.

## Data Availability

The original contributions presented in the study are included in the article, further inquiries can be directed to the corresponding author.

## Supplementary Materials

The following information can be downloaded at https://www.mdpi.com/article/10.3390/foods13162609/s1, Figure S1: HPLC chromatogram of sugar syrup made using unripe *maesil* pulp (U1) at 6 months; Figure S2: HPLC chromatogram of sugar syrup from which unripe *maesil* was removed after three months (U2) at 6 months; Figure S3: HPLC chromatogram of sugar syrup made using unripe whole *maesil* (U3) at 6 months; Figure S4: HPLC chromatogram of sugar syrup made using ripe *maesil* pulp (R1) at 6 months; Figure S5: HPLC chromatogram of sugar syrup from which ripe *maesil* was removed after three months (R2) at 6 months; Figure S6: HPLC chromatogram of sugar syrup containing ripe whole *maesil* (R3) at 6 months.
