# Supplementary material for: The Effect of One-Year Fermentation of Maesil Fruit (Prunus mume) Sugar Syrup on Amygdalin Level: A Natural Toxic Compound
Source: Foods. 2024 Aug 20;13(16):2609. doi: 10.3390/foods13162609 (PMC11353800; doi:10.3390/foods13162609)
Supplement: Supplementary file 1 [file foods-13-02609-s001.zip › foods-3077544-supplementary.pdf]

The Effect of One-Year Fermentation of *Maesil* Fruit (*Prunus mume*) Sugar Syrup on Amygdalin Level: A Natural Toxic Compound

Srinivasan Ramalingam [1,†], Vishal Kumar [1,†], Ashutosh Bahuguna [1], Jong Suk Lee [2] and Myunghee Kim [1,3,*]

[1] Department of Food Science and Technology, Yeungnam University, Gyeongsan 38541, Republic of Korea; sribt27@gmail.com (S.R.); vishalkumar@yu.ac.kr (V.K.); ashubahuguna@ynu.ac.kr (A.B.)

[2] Department of Food & Nutrition & Cook, Taegu Science University, Daegu 41453, Republic of Korea; jslee1213@ynu.ac.kr

[3] Research Institute of Cell Culture, Yeungnam University, Gyeongsan 38541, Republic of Korea

* Correspondence: foodtech@ynu.ac.kr

† These authors contributed equally to this work.

Supplementary data

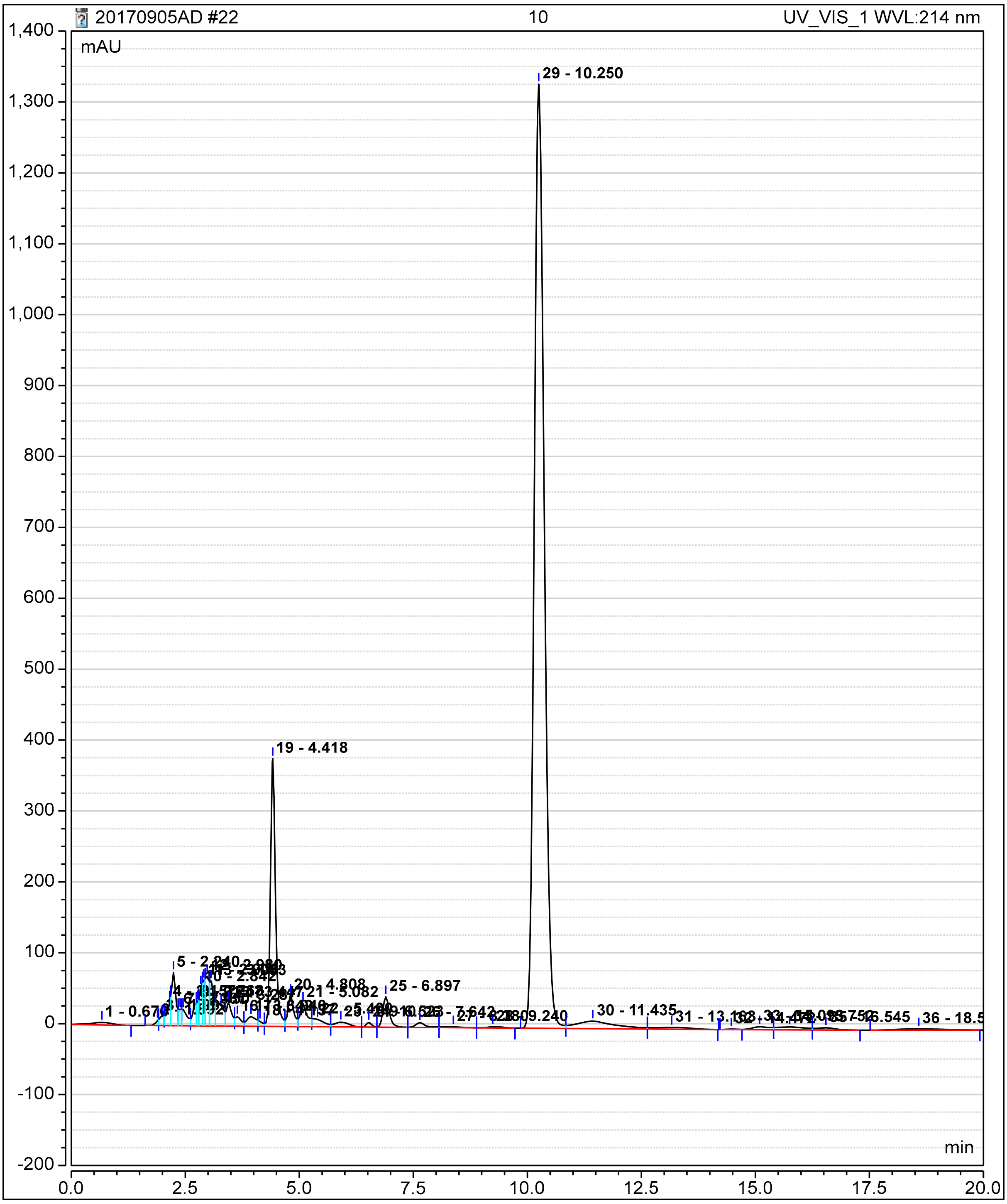

Supplementary Figure S1. HPLC chromatogram of sugar syrup made using unripe *maesil* pulp (U1) at 6 months.

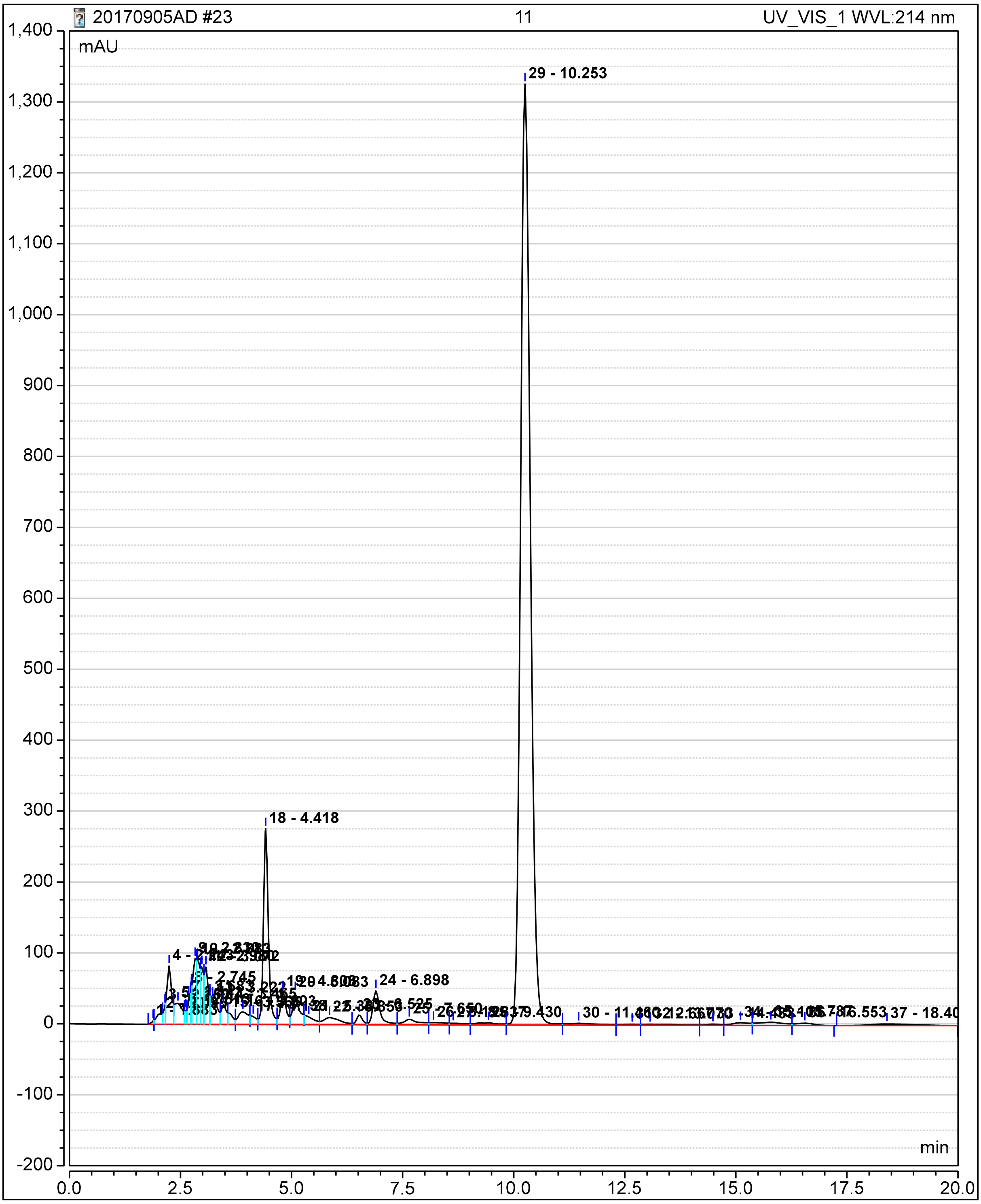

Supplementary Figure S2. HPLC chromatogram of sugar syrup from which unripe *maesil* was removed after three months (U2) at 6 months.

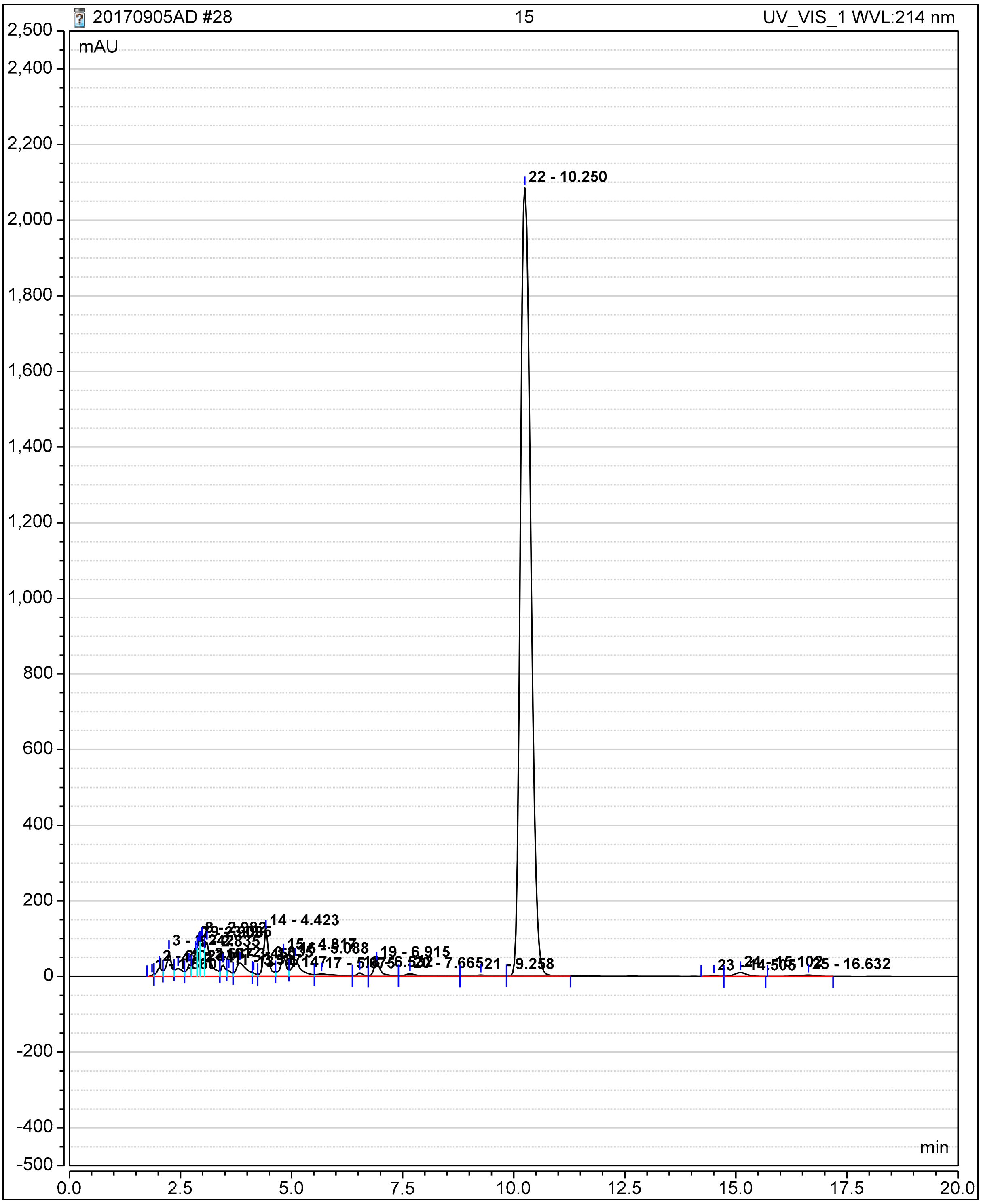

Supplementary Figure S3. HPLC chromatogram of sugar syrup made using unripe whole *maesil* (U3) at 6 months.

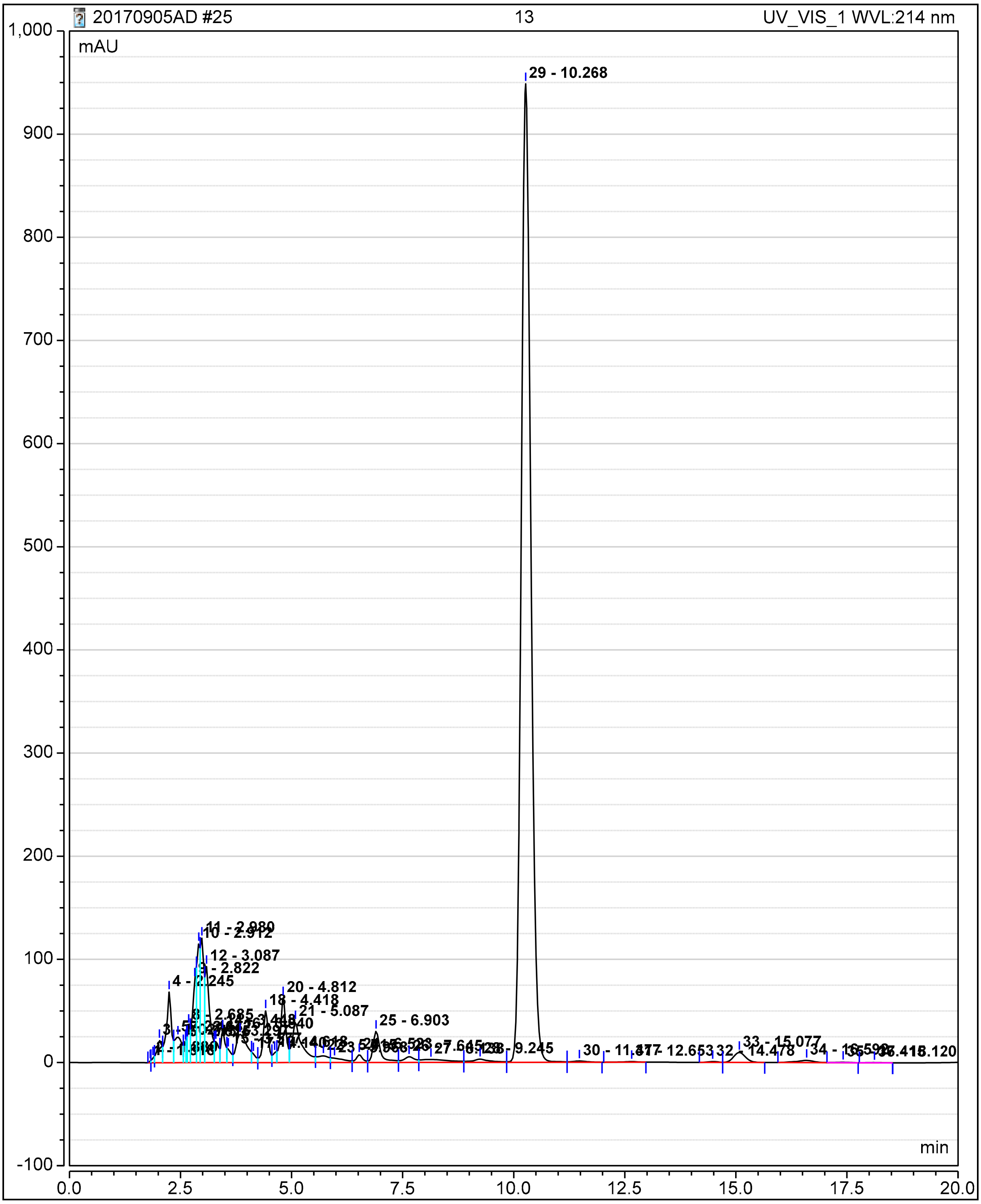

Supplementary Figure S4. HPLC chromatogram of sugar syrup made using ripe *maesil* pulp (R1) at 6 months.

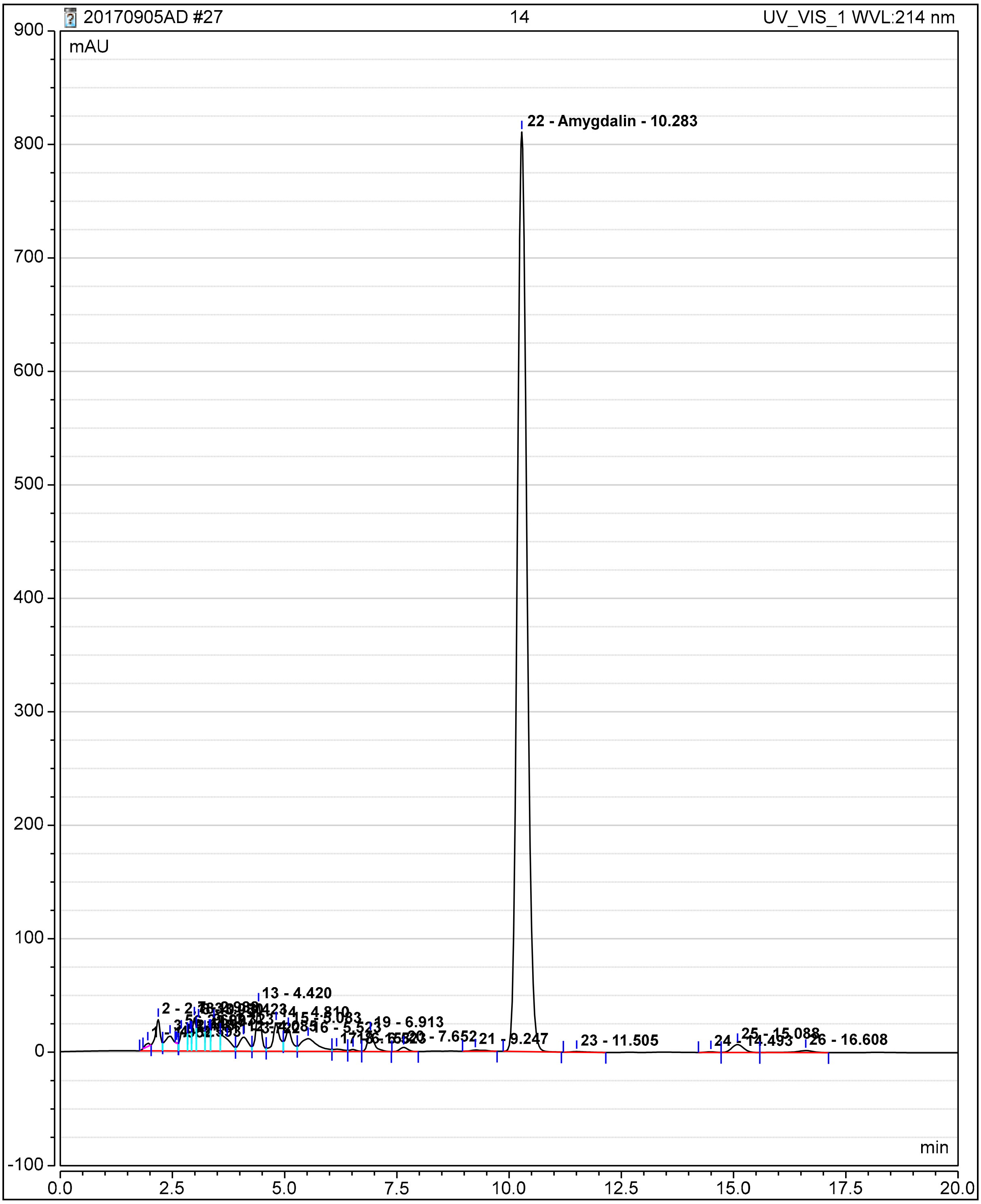

Supplementary Figure S5. HPLC chromatogram of sugar syrup from which ripe *maesil* was removed after three months (R2) at 6 months.

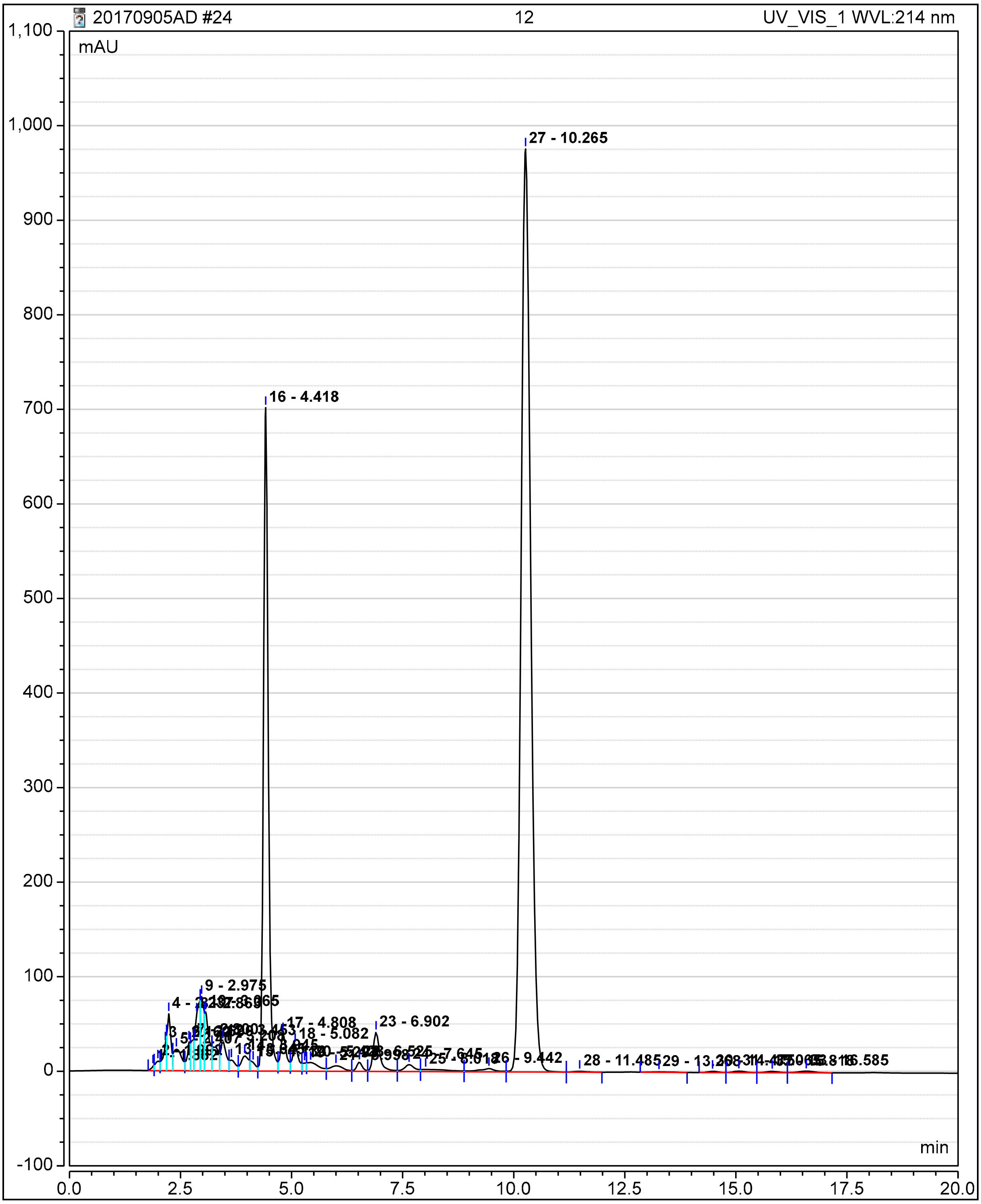

Supplementary Figure S6. HPLC chromatogram of sugar syrup containing ripe whole *maesil* (R3) at 6 months.
